# Development and Optimization of a Recyclable Non-Embedded Support System for Thermal Pipeline Trenches in Urban Environments

**DOI:** 10.3390/ma18010068

**Published:** 2024-12-27

**Authors:** Jianfei Ma, Shaohui He, Gangshuai Jia

**Affiliations:** School of Civil Engineering, Beijing Jiaotong University, Haidian District, Beijing 100044, China; 22110333@bjtu.edu.cn (J.M.); 22121080@bjtu.edu.cn (G.J.)

**Keywords:** thermal engineering, trench support, prototype test, numerical simulation, field application

## Abstract

Existing support systems for thermal pipeline trenches often fail to meet the specific needs of narrow strips, tight timelines, and short construction periods in urban environments. This study introduces a novel recyclable, non-embedded support system composed of corrugated steel plates, retractable horizontal braces, angle steel, and high-strength bolts designed to address these challenges. The system’s effectiveness was validated through prototype testing and optimized using Abaqus finite element simulations. The research hypothesizes that this new support structure will enhance construction efficiency, reduce installation costs, and provide adaptable and sustainable solutions in urban trench applications. Prototype tests demonstrated that the proposed support had maintained safety and stability in trenches of 2 m and 3 m depth under a 58 kPa load and rainfall, as well as the 4 m deep trenches under asymmetric loading of 80 kPa. Optimization of the proposed system included installing two screw jacks on each horizontal brace and adjusting the corrugated plates, resulting in reduced weight, improved node strength, and enhanced screw jack adjustability. Numerical simulations confirmed the optimized system’s reliability in trenches up to 3 m deep, with caution required for deeper applications to avoid structural failure. The proposed support system offers notable advantages over traditional methods by improving construction efficiency, flexibility, and adaptability while also reducing costs, ensuring safety, and promoting environmental sustainability. Its modular design allows for rapid installation and disassembly, making it suitable for projects with strict deadlines and diverse construction conditions. The findings uphold the initial hypotheses and demonstrate the system’s practicality in urban trench projects.

## 1. Introduction

With the rapid pace of urbanization, the demand for the construction, maintenance, and renovation of underground pipelines in urban sensitive areas has grown dramatically [1]. For instance, over 100,000 km of underground pipelines are expected to be renovated annually in China in the coming years. Trenching is required for the installation, emergency repairs, and upgrades of pipelines. Given the small excavation volumes and short construction durations of pipeline foundation pits, excavation is often carried out by sloping or using simple support structures [2]. However, this approach increases the risk of trenches (or foundation pits)overturning and collapse accidents [3,4]. Traditional excavation shoring systems, such as steel sheet piles, soil-nailing walls, and timber supports, have been commonly used for supporting trench walls and preventing collapse. These systems, while effective in certain cases, often face limitations in terms of cost, adaptability, and environmental impact. Developing a new support system tailored to the unique requirements of underground pipeline projects is a pressing challenge in the field of municipal engineering.

To date, the construction and stability of pipeline trenches and foundation pits have garnered significant attention from scholars [5,6]. For example, Dang and Zhang [7] optimized the design and construction of internal support structures for foundation pits based on an actual construction project. Using the numerical simulation, Wang et al. [8] explored the support effects of soil-nailing walls when pipelines are affected by foundation pit construction, analyzing four factors: excavation depth, pipeline burial depth, the distance between excavation area and pipeline, and soil strength. Aiming to reduce the disturbance of tunneling and deep foundation pits to adjacent buried pipelines, Zhang et al. [9] derived an analytical solution for pipeline deformation in clay based on the Mindlin solution. Later, Zhang et al. [10] established analytical and three-dimensional models to investigate the deformation of pipelines and foundation pits, finding that underground continuous walls could effectively reduce pipeline deformation. The authors of Jiang et al. [11] proposed a predictive model of stress for pipelines under various operational conditions to evaluate pipeline safety considering foundation pit blasting construction. In summary, most related studies focus on construction disturbance effects, construction standards, deformation control solutions, risk state assessments, and countermeasures for deep foundation pits and tunnels adjacent to pipelines [12,13,14,15]. However, foundation pits (or trenches) for pipelines, including power, water supply, and heat pipelines, typically feature small excavation volumes, narrow strip types, short construction periods, and shallow burial depths. The safety of these small-scale foundation pits (or trenches) has not received adequate attention, as evidenced by numerous accidents recorded in recent years. Commonly used support structures in urban pipeline foundation pits include steel sheet piles, soil-nailing walls, timber supports, and cast-in-situ concrete supports. In this regard, Ouyang et al. [16] introduced the Gauss–Legendre method to calculate the interaction between steel sheet piles and the surrounding soil. The authors of Dhutti et al. [17] addressed steel sheet pile corrosion issues by proposing the use of the GWT method to detect movement zones in steel sheet piles. The authors of Gao et al. [18] conducted model tests to analyze the stress characteristics of steel sheet piles. The authors of Jiang et al. [19], using a cofferdam project as a case study, utilized numerical simulations to analyze the stability of double-row steel sheet pile structures in soft soil foundations. These studies primarily focus on evaluating the stability and reliability of support structures, as well as the effectiveness of new support structure applications. However, existing research on urban underground pipelines fails to accurately reflect the specific characteristics of foundation pit construction in municipal engineering. The authors of Shao et al. [2] noted that shallow foundation pits for power cables often suffer from either overly simplistic and inadequate support or excessive use of steel sheet piles, leading to construction risks and support redundancy.

Therefore, this paper proposes a novel recyclable, non-embedded support system composed of corrugated steel plates, retractable inner braces, angle steels, and high-strength bolts. The hypothesis of this research is that this proposed support will provide an efficient, cost-effective, and sustainable solution for urban pipeline trenching, improving both the speed and safety of construction while minimizing environmental impact. A prototype test was conducted to verify its mechanical performance and practical effectiveness. Additionally, numerical simulations were employed to optimize the structure. The system is designed to address urban challenges like limited space, tight deadlines, and the need for eco-friendly solutions. This study’s findings are expected to impact municipal engineers, urban planners, and construction managers, especially in underground pipeline construction and maintenance. The proposed support system offers a flexible, sustainable solution with international application, particularly in rapidly urbanizing regions.

## 2. Recyclable Non-Embedded Support Design

### 2.1. Characteristics of Heat Pipeline Trench Support

**Figure 1:**
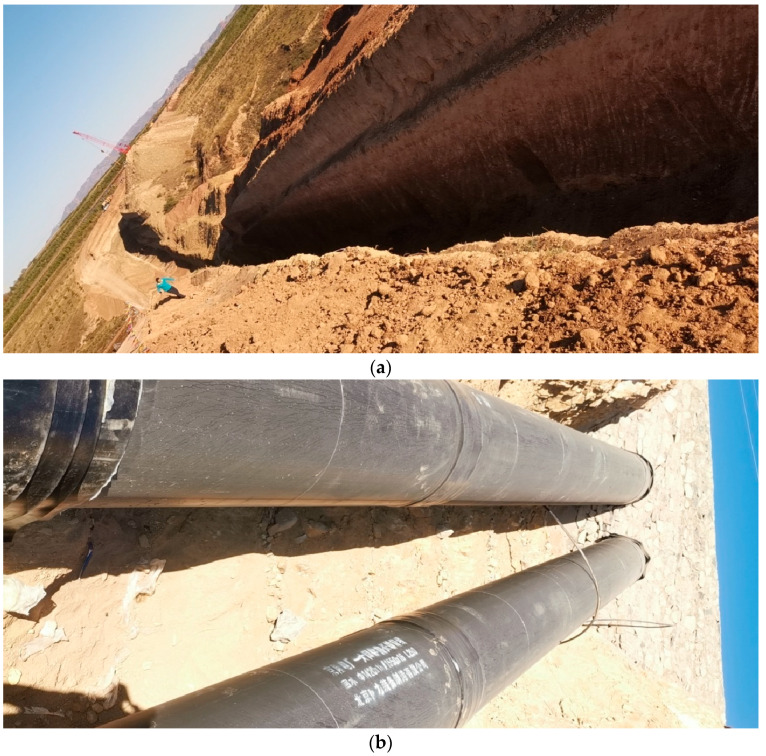
Thermal trench construction site; (**a**) trench; (**b**) pipeline.

The support structure for thermal pipeline trenches, as shown in Figure 1, should fulfill the following requirements:

(1) Flexibility and adjustability: To meet the needs of varying construction conditions and unforeseen circumstances.
(2) Construction efficiency and cost-effectiveness: To shorten the construction period and reduce project costs.
(3) Consideration of pipeline construction impacts: To ensure that the support structure does not impede the smooth installation of pipelines within the trench.

### 2.2. Problems in the Current Support System

Through onsite surveys, engineering comparisons, and literature analysis, the following problems were identified in the current thermal pipeline trench construction:

(1) Unsatisfactory support effectiveness: Some projects lacked adequate support or employed simplistic support methods. In particular, under soft soil or complex geological conditions, trench collapse can easily occur, posing substantial safety risks. Additionally, some projects utilized excessively robust support structures (such as oversized steel sheet piles or concrete supports), leading to resource wastage and increased costs without optimizing the benefits of the support.
(2) Poor timeliness and limited applicability: Traditional support methods (such as steel sheet piles or concrete diaphragm piles) involve complex construction processes and extended timelines, making them unsuitable for emergency repairs or short-term projects. Furthermore, many support structures rely on specialized equipment (such as vibratory hammers and drilling rigs), adding complexity to the construction site. Most traditional support forms are standardized designs, which struggle to accommodate irregularly shaped trenches or complex terrains, thereby limiting their applicability.
(3) Poor economic efficiency: The materials used in existing support structures are not reusable, resulting in long-term material waste and high financial investments. Current designs are not conducive to modular or assembly-based construction, leading to poor adaptability and reduced economic benefits. Moreover, difficulties in disassembly result in low recycling rates of support structures, further escalating construction costs.
(4) High carbon emissions: The production, transportation, and installation of steel and concrete involve significant energy consumption, contributing to considerable carbon emissions. Additionally, the use of high-power equipment during construction further increases the carbon footprint.

### 2.3. Support Design of Recyclable Non-Embedded Support Design

**Figure 2:**
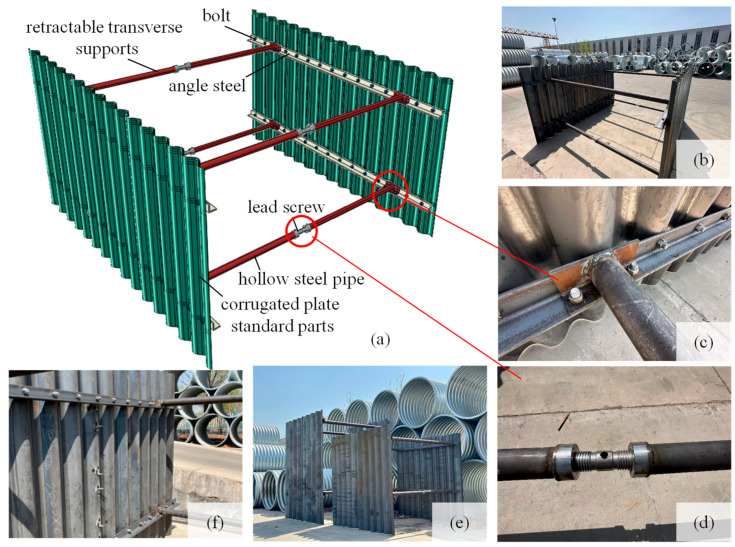
New support structure; (**a**) structure diagram; (**b**) 2 m high structure; (**c**) corrugated plate-horizontal support joint; (**d**) lead screw; (**e**) 3 m and 4 m high structure; (**f**) corrugated plate.

Addressing characteristics of narrow strip type, shallow depth, and high timeliness requirement for the trench of the thermal pipeline, a recyclable support structure without embedment depth was proposed, as shown in Figure 2. The support structure comprises corrugated plate structural standard parts, retractable transverse supports (i.e., horizontal braces), angle steel, and high-strength bolts. Compared to flat plates, corrugated steel standard parts offer large out-of-plane stiffness and high buckling strength [20]. The corrugated plate standard parts are connected by angle steel after overlapping along the trench axis. A lead screw is installed in the center of the retractable strut, allowing adjustment of the spacing of corrugated steel on both sides. Angle steel and corrugated plates, as well as angle steel and transverse supports, are connected using high-strength bolts.

### 2.4. Construction Technology of the Proposed Support

**Figure 3:**
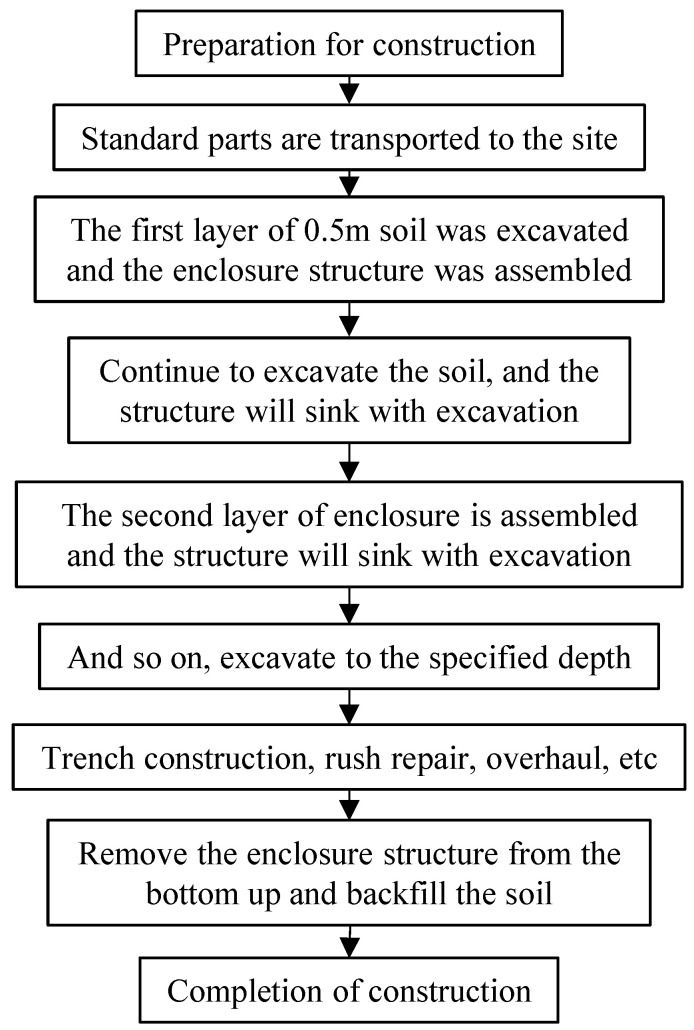
Construction technology of recyclable non-embedded support structure.

The construction technology for the recyclable non-embedded support structure, suitable for the construction of narrow strip trenches for thermal pipelines, is illustrated in Figure 3.

(1) Preparation and Transportation: After the standard parts of the proposed support are calibrated and assembled by the manufacturer, they are transported to the trench construction area and handed over to the on-site construction personnel.
(2) Initial Excavation and Installation: Small-scale excavation machinery is used to excavate the soil in the designated area, with an initial excavation depth of 0.5 m. Upon completion of the first layer of soil excavation, the lightweight envelope structure is installed. The flexible supports are adjusted using lead screws, ensuring that the corrugated plates are closely attached to the soil behind the supports. Excavation then continues with single-layer depths not exceeding 0.2 m, allowing the lightweight envelope structure to sink progressively with the excavation.
(3) Support Installation: When the excavation depth reaches a standard section height, the second layer of standard sections is installed. Excavation continues downward with single-layer depths not exceeding 0.2 m, and the support structure is lowered accordingly. This process is repeated until the trench is excavated to the specified depth.
(4) Pipeline Installation and Construction Activities: The internal pipeline is buried within the trench, and any necessary emergency repairs or other engineering construction activities are carried out.
(5) Deinstallation and Backfilling: The lowest supporting structure is removed, and the excavated soil is backfilled. Subsequently, the second-to-last layer of supports is removed, and the soil is backfilled. This process continues iteratively until all support enclosures are removed, the soil is fully backfilled, and construction is completed.

## 3. Prototype Test of New Thermal Trench Support

### 3.1. Test Site and Support Design

The prototype test site is located in the central and southern plains of Hebei Province, with an altitude of 15.10~22.50 m and an average altitude of 20 m. The soil in the test area is predominantly silt. The physical and mechanical parameters of soil in the test area are obtained by the triaxial compression test and the elastic modulus test. The physical and mechanical properties of the soil were determined through triaxial compression tests and elastic modulus tests. The silt has a dry density of 1.33 g/cm^3^ and a water content of 8.8%. The average elastic modulus is 18.82 MPa, Poisson’s ratio is 0.19, cohesion is 1.7 kPa, and the internal friction angle is 24.48°.

**Figure 4:**
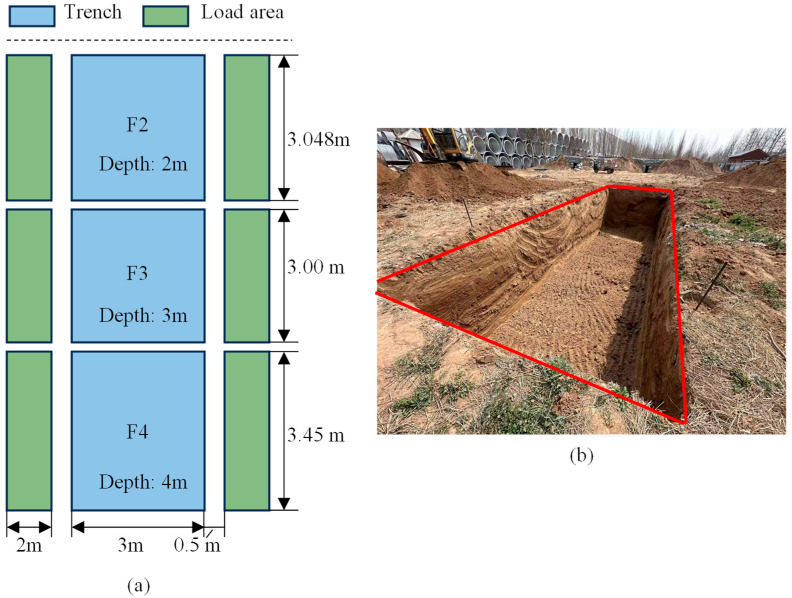
Test site diagram; (**a**) test site diagram; (**b**) test site.

Three trenches (designated F2, F3, F4) were constructed for the prototype test. Each trench has a width of 3 m. The depths and lengths of the trenches are as follows: trench F2 has a depth of 2 m and a length of 3.048 m, trench F3 has a depth of 3 m and a length of 3 m, and trench F4 has a depth of 4 m and a length of 3.45 m, as shown in Figure 4. The loading areas are located on both the left and right sides of each trench, positioned 0.5 m from the trench edges. Each loading area measures 2 m in length and 3 m in width, covering an area of approximately 6 m². The design parameters of the supporting structure are listed in Table 1.

### 3.2. Test Scheme and Loading Design

Four test cases (designated as F2, F3, F2_R, and F4) were conducted in the prototype test, as summarized in Table 2. Case F2 applies symmetric loading at incremental load levels of 10, 18, 26, 34, 42, 50, and 58 kPa without any rainfall. Case F3 utilizes the same load levels as F2 but applies them asymmetrically, also without rainfall. Case F2_R involves a symmetric load of 58 kPa and includes simulated rainfall. Case F4 applies asymmetric loading at 10, 30, 50, and 80 kPa with no rainfall.

**Figure 5:**
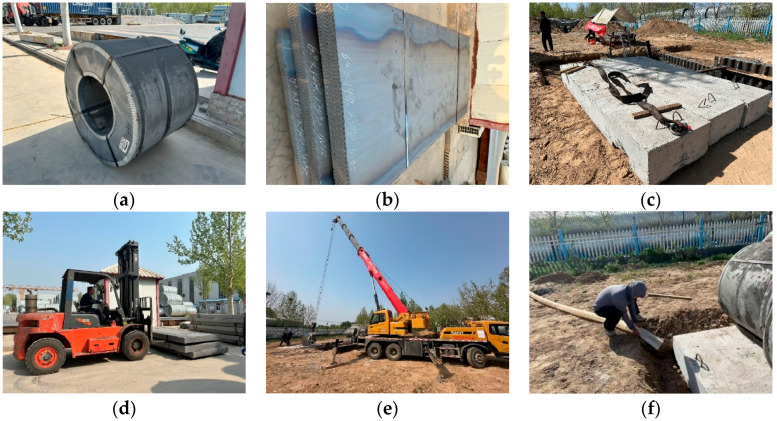
Test loading equipment; (**a**) steel coils; (**b**) steel plates; (**c**) loading base; (**d**) forklift; (**e**) crane; (**f**) rainfall simulation.

Galvanized steel coils and galvanized steel plates were used as loading weights, with a concrete plate serving as the loading base. Each steel coil weighs 5 t, and a total of 12 coils were prepared. Each steel plate weighs 0.213 t, with a total of 24 t prepared. During the experiment, a forklift was used to transport the steel coils and plates to the test site, and a crane was employed to position them. Rainfall was simulated by introducing water into the trench, with a maximum simulated rainfall of 50 mm (to replicate the test area’s maximum rainfall event in the past 6 months), as shown in Figure 5.

### 3.3. Monitoring System

**Figure 6:**
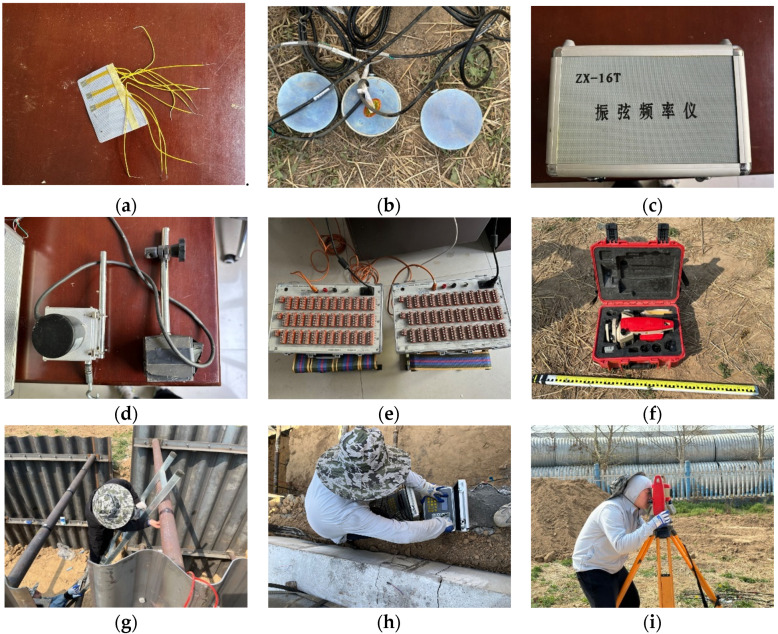
Test equipment and its testing site; (**a**) strain gauge; (**b**) earth pressure box; (**c**) frequency meter; (**d**) cable displacement sensor; (**e**) static stress and strain testing and analysis system; (**f**) total station; (**g**) strain gauge installment; (**h**) earth pressure measurement; (**i**) soil settlement measure.

**Figure 7:**
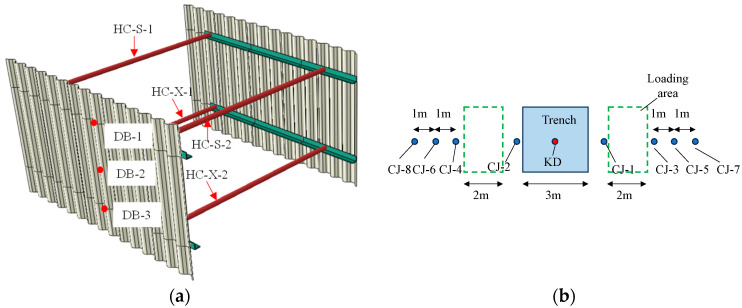
Monitoring point layout; (**a**)stress monitoring points; (**b**) settlement monitoring points.

The monitoring parameters primarily include: (1) the stress and deformation of the support structure; (2) earth pressure behind the corrugated plate; and (3) soil settlement. The monitoring instruments utilized in this study are as follows: 120-5AA strain gauge, DH3816N static stress and strain testing and analysis system, XJM surface strain gauge, ZX-16T vibrating string frequency meter, steel string earth pressure sensor, WS-S-100 mm cable displacement sensor, KTS-442U total station, etc. A portion of the test equipment and the testing site setup are illustrated in Figure 6. The monitoring points in the prototype are shown in Figure 7.

## 4. Prototype Test Results of the New Non-Embedded Support

### 4.1. Observed Test Phenomenon

**Figure 8:**
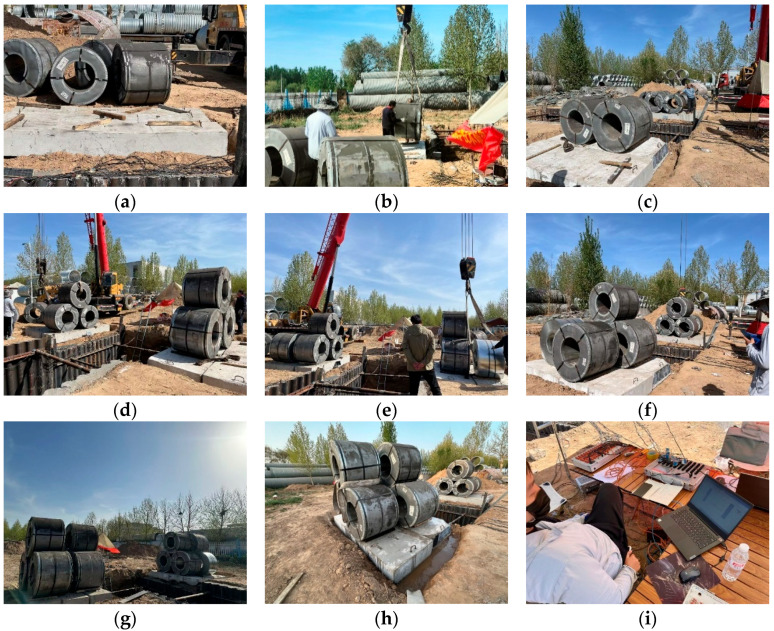
Prototype test phenomenon; (**a**) loading base; (**b**) first steel coil; (**c**) second steel coil; (**d**) third steel coil; (**e**) fourth steel coil; (**f**) fifth steel coil; (**g**) sixth steel coil; (**h**) rain simulation; (**i**) monitoring site.

The prototype test site for the new thermal trench support is illustrated in Figure 8, using conditions F2 and F2_R as examples. In tests F2 and F3, a 50 kPa load was applied to both sides of the trench. Regardless of whether the loading was asymmetric or symmetric, no damage occurred to the support structure, and both the structure and the surrounding soil remained stable. Even under the combined action of a 50 kPa load and rainfall (condition F2_R), the support structure experienced no damage, maintaining overall stability in both the structure and the trench. In the F4 test, after applying an 80 kPa load at the trench’s edge, the support structure remained stable. These experimental observations demonstrate that, under the current design parameters, the proposed support system can withstand loads exceeding 50 kPa—more than twice the vehicle load requirement specified by Chinese standards.

### 4.2. Structure Deformation Analysis of Prototype Test

**Figure 9:**
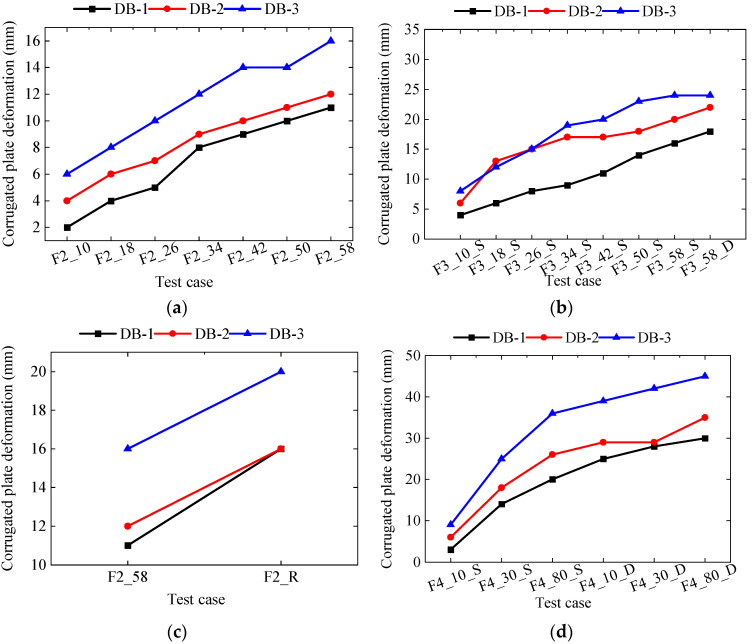
Relative deformation of a corrugated plate; (**a**) F2 case; (**b**) F3 case; (**c**) F2_R case; (**d**) F4 case.

The relative deformation of the corrugated plates in the prototype test is illustrated in Figure 9. Under condition F2, a 58 kPa load was applied symmetrically to both sides of the trench, resulting in maximum deformations of 11 mm, 12 mm, and 16 mm at measurement points DB-1, DB-2, and DB-3, respectively. In condition F3, the same 58 kPa load was applied asymmetrically to both sides of the trench, leading to maximum deformations of 18 mm, 22 mm, and 24 mm at DB-1, DB-2, and DB-3, respectively. Under condition F2_R, a 58 kPa load combined with simulated rainfall was applied symmetrically to both sides of the trench, resulting in maximum deformations of 16 mm, 16 mm, and 22 mm at DB-1, DB-2, and DB-3, respectively. The introduction of rainfall increased the deformation of the corrugated plates by 45.45%, 33.33%, and 25.00% at DB-1, DB-2, and DB-3, respectively. In condition F4, an 80 kPa load was applied asymmetrically to both sides of the trench, resulting in maximum deformations of 30 mm, 35 mm, and 45 mm at DB-1, DB-2, and DB-3, respectively. Overall, as the applied load increased, the relative deformation of the corrugated plates on both sides of the trench also increased. Additionally, deeper trenches exhibited greater relative deformation of the corrugated plates.

The maximum deformation of the transverse support structure in the prototype experiment is depicted in Table 3.

(1) Case F2: Under this condition, a symmetrical load of 58 kPa was applied to both sides of the trench. The deformations recorded at measurement points HC-X-1, HC-X-2, HC-S-1, and HC-S-2 were 4.0 mm, 3.0 mm, 3.0 mm, and 3.0 mm, respectively.
(2) Case F3: In this scenario, a 58 kPa load was applied to one side of the trench, resulting in deformations of 2.0 mm, 3.0 mm, 2.0 mm, and 2.0 mm at points HC-X-1, HC-X-2, HC-S-1, and HC-S-2, respectively. When the same symmetrical 58 kPa load was applied to both sides, the deformations increased to 4.0 mm, 5.0 mm, 3.0 mm, and 3.0 mm, respectively.
(3) Case F2_R: This test involved a symmetrical load of 58 kPa combined with simulated rainfall. The deformations observed were 6.0 mm, 7.0 mm, 5.0 mm, and 5.0 mm at HC-X-1, HC-X-2, HC-S-1, and HC-S-2, respectively. The introduction of rainfall resulted in deformation increases of 50.00%, 40.00%, 66.67%, and 66.67% at these points, respectively.
(4) Case F4: Under this condition, an 80 kPa load was applied asymmetrically to one side of the trench, leading to deformations of 3.0 mm, 3.0 mm, 2.0 mm, and 2.0 mm at HC-X-1, HC-X-2, HC-S-1, and HC-S-2, respectively. When the same load was applied symmetrically to both sides, the deformations increased to 6.0 mm, 7.0 mm, 4.0 mm, and 4.0 mm, respectively.

Overall, the results indicate that as the applied load on both sides of the trench increased, the deformation of the transverse support structures also increased. Additionally, deeper trenches exhibited greater relative deformation of the transverse support structures.

### 4.3. Structure Stress Analysis of Prototype Test

**Figure 10:**
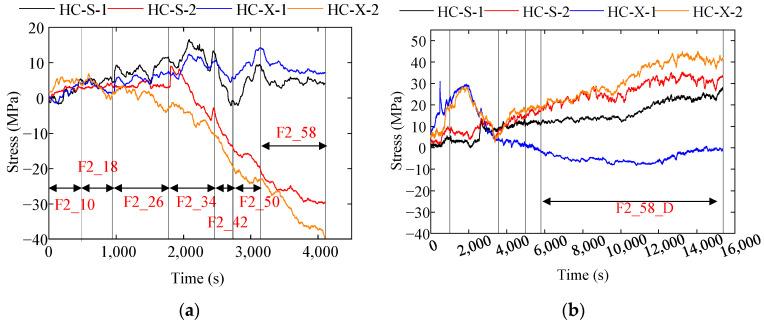
Stress time history curve; (**a**) transverse support stress in a 2 m deep trench; (**b**) transverse support stress in a 3 m deep trench.

**Figure 11:**
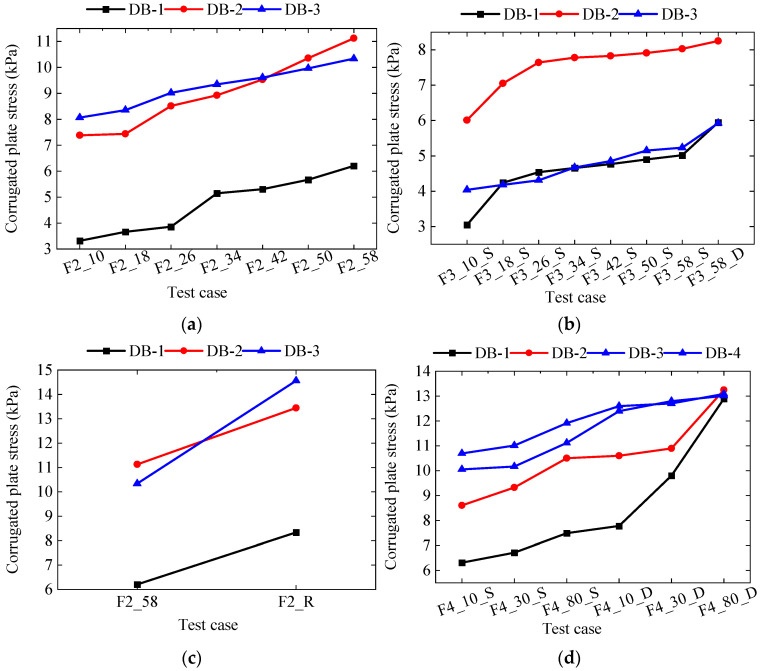
Stress of corrugated plate; (**a**) F2 case; (**b**) F3 case; (**c**) F2_R case; (**d**) F4 case.

The stress–time history curve of the structure in the prototype test is shown in Figure 10, using transverse support stress as an example. The maximum stresses of the corrugated plates and transverse support at each test stage are illustrated in Figure 11 and Table 4.

(1) Case F2: Under this condition, a symmetrical load of 58 kPa was applied to both sides of the trench. The maximum stresses recorded at measurement points DB-1, DB-2, and DB-3 for the corrugated plates were 6.21 MPa, 11.13 MPa, and 10.34 MPa, respectively. The maximum stress in the transverse support structure was −30.13 MPa.
(2) Case F3: In this scenario, a symmetrical load of 58 kPa was applied to both sides of the trench. The maximum stresses at DB-1, DB-2, and DB-3 measurement points of the corrugated plates were 5.95 MPa, 8.25 MPa, and 5.93 MPa, respectively. The maximum stress in the transverse support structure was 41.57 MPa.
(3) Case F2_R: This test involved a symmetrical load of 58 kPa combined with simulated rainfall. The maximum stresses observed at DB-1, DB-2, and DB-3 measurement points of the corrugated plates were 8.34 MPa, 13.45 MPa, and 14.56 MPa, respectively. The maximum stress in the transverse support structure was −61.51 MPa. The introduction of rainfall resulted in deformation increases of 50.00%, 40.00%, 66.67%, and 66.67% at these points, respectively.
(4) Case F4: Under this condition, a symmetrical load of 80 kPa was applied to both sides of the trench. The maximum stresses recorded at DB-1, DB-2, and DB-3 measurement points of the corrugated plates were 12.9 MPa, 13.25 MPa, and 13.09 MPa, respectively. The maximum stress in the transverse support structure was −13.30 MPa.

In summary, when the new support structure was applied to thermal trenches with depths of 2 m, 3 m, and 4 m, the maximum stress in the support structure was significantly lower than the yield strength of the materials used, and no structural damage occurred.

### 4.4. Soil Settlement Analysis

**Figure 12:**
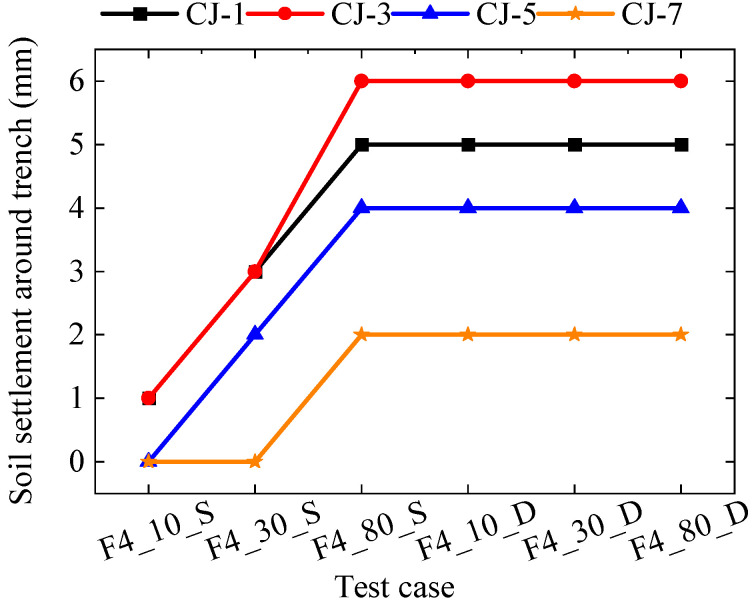
Soil settlement around the trench.

The deformation of the soil surrounding the trench and at the trench bottom in the prototype test of the new support structure is illustrated in Figure 12 and Table 5, taking a trench with a depth of 4 m as an example (i.e., Case F4). During the test, as the weight of the unilateral load on the trench increased, the settlement of the external soil gradually increased. Upon completion of the unilateral loading, the maximum settlements at measurement points CJ-1, CJ-5, and CJ-7 were 5 mm, 6 mm, and 4 mm, respectively. Bilateral loading had minimal impact on surface settlement. Following the completion of the test loading, the maximum uplift deformation of the trench bottom soil was 4 mm.

From a comprehensive analysis of structural deformation, stress, and soil deformation, the in situ test results indicate that the support structure did not suffer any damage, and both the structure and soil remained stable. The new support structure demonstrated excellent performance in the thermal pipeline trench.

## 5. Optimization Design of Non-Embedded Support

### 5.1. Optimization Design Scheme

Based on the findings from the prototype tests, the following issues were identified:

(1) Excessive weight of the support: the current support structure is slightly heavy, with a weight that still exceeds that of lightweight structures;
(2) Limited adjustment margin of the horizontal support screw: The existing design restricts the range of adjustment for the horizontal support screws.
(3) Suboptimal screw placement: The screws are positioned at the least optimal stress points in the middle of the horizontal supports, making them susceptible to jamming.

To address these issues, the non-embedded support system was optimized with the following modifications:

(1) Enhanced screw installation: Screws are now installed on each horizontal support, with two screws per structure. Additionally, the screws have been relocated closer to the corrugated plates to improve stability and reduce the risk of jamming.
(2) Weight reduction: The parameters of the corrugated plates, angle steel, and horizontal supports have been reduced to decrease the overall weight of the support structure. These changes are detailed in Table 6.

### 5.2. Geotechnical–Structural Model

#### 5.2.1. Limitations and Simplifications in Geotechnical Assumptions

The numerical model in this paper is based on the following key assumptions:

(1) It is assumed that dewatering has been completed prior to the construction of the trench. Consequently, the model does not account for the influence of groundwater or the effects of underground water on soil behavior during construction.
(2) The materials in the model are assumed to be homogeneous and isotropic for simplification. This assumption does not take into account potential variations in material properties, such as spatial heterogeneity or anisotropy, which could affect the accuracy of the results in real-world scenarios.
(3) The model does not incorporate the effects of soil consolidation or settlement behavior over time, which could influence the long-term performance and stability of the support structure.
(4) Additionally, select sandy soil (which is one of the most common soil types in the shallow layers of the North China Plain and also represents the soil type found in the in situ test area) as the soil material for the numerical model.

#### 5.2.2. Establishing Numerical Model

A non-linear geotechnical–structural model of the thermal pipeline trench support structure was developed using Abaqus (Version 2022) finite element analysis to investigate the mechanical performance of the optimized support system. The model dimensions are 27 m in length, 3.45 m in width, and 10 m in height. The support structure itself is 3 m wide and is positioned 12 m (four times the width of the support structure) from the boundary on both sides. Choosing the 2 m deep structure as an example, the maximum mesh size of soil, corrugated steel plate, transverse support, screw, angle steel, and bolt are 100, 10, 20, 5.0, 3.5 and 2.8 mm, respectively. These mesh sizes were optimized to balance computational efficiency and accuracy. The element type used for meshing is C3D8R, which is an 8-node linear brick element with reduced integration and hourglass control.

**Figure 13:**
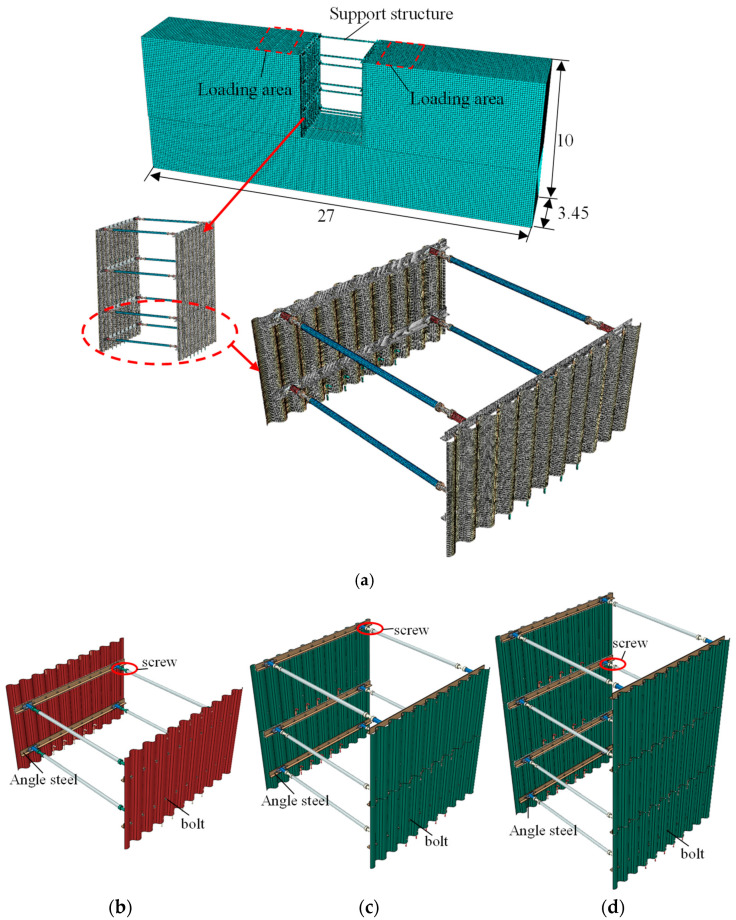
Optimized model; (**a**) numerical model; (**b**) support for 2 m deep trench; (**c**) support for 3 m deep trench; (**d**) support for 4.5 m deep trench.

In the model, the support structure is constructed from Q345 steel and employs an elastic constitutive model. It is assumed that the material enters a plastic state once the applied stress exceeds its yield strength. The surrounding soil is modeled using the Mohr–Coulomb constitutive model, with soil parameters matching those of the in situ test layer. The model is constrained to prevent normal displacement around its perimeter, featuring fixed boundary conditions at the bottom and a free surface at the top. To address the issue of convergence between different mesh sizes, it is assumed that the angle steel is rigidly connected to the corrugated plate, utilizing the Tie algorithm for modeling. The bolts connecting the horizontal support and angle steel to the corrugated plate, as well as the soil-corrugated plate interaction, are modeled using surface-to-surface contact interactions. Since there are no specific parameters defined in current experimental standards and engineering codes for heat conduction trenches, a load equivalent to twice the vehicle load (i.e., 40 kPa) is applied to both sides of the trench, referring to Chinese Code for Design of Building Foundation (GB 50007-2011) [21]. The numerical simulation model is depicted in Figure 13a, which includes trench depths of 2 m, 3 m, and 4.5 m, as illustrated in Figure 13b–d.

### 5.3. Numerical Simulation Results

#### 5.3.1. Structure Stress Analysis

**Figure 14:**
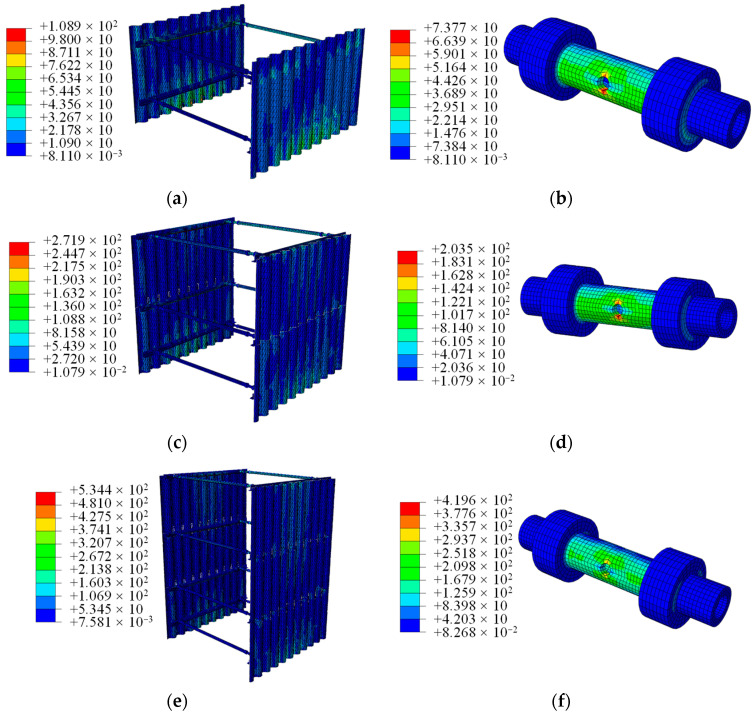
Structural stress nephogram; (**a**) structural stress in 2 m deep trench; (**b**) screw stress in 2 m deep trench; (**c**) structural stress in 3 m deep trench; (**d**) screw stress in 3 m deep trench; (**e**) structural stress in 4.5 m deep trench; (**f**) screw stress in 4.5 m deep trench.

After applying twice the vehicle load to both sides of the thermal pipeline trench, the stress distribution of the support structure is depicted in Figure 14, illustrating both the overall stress of the structure and the stress within the screw jacks. Generally, the maximum stress within the entire support structure occurs at the upper corner steel. The highest stress in the screw jacks is observed at the hole locations, while the maximum stress in the bolts occurs at the bolt-to-angle steel contact surfaces.

At trench depths of 2 m, 3 m, and 4.5 m, the maximum stresses in the structure are 108.9 MPa, 271.9 MPa, and 534.4 MPa, respectively. In summary, the optimized support structure remains safe and stable when applied to trenches with depths of 2 m and 3 m. However, when applied to a trench with a depth of 4.5 m, the structural stress exceeds the material’s yield strength, potentially leading to structural failure.

#### 5.3.2. Structure Deformation Analysis

**Figure 15:**
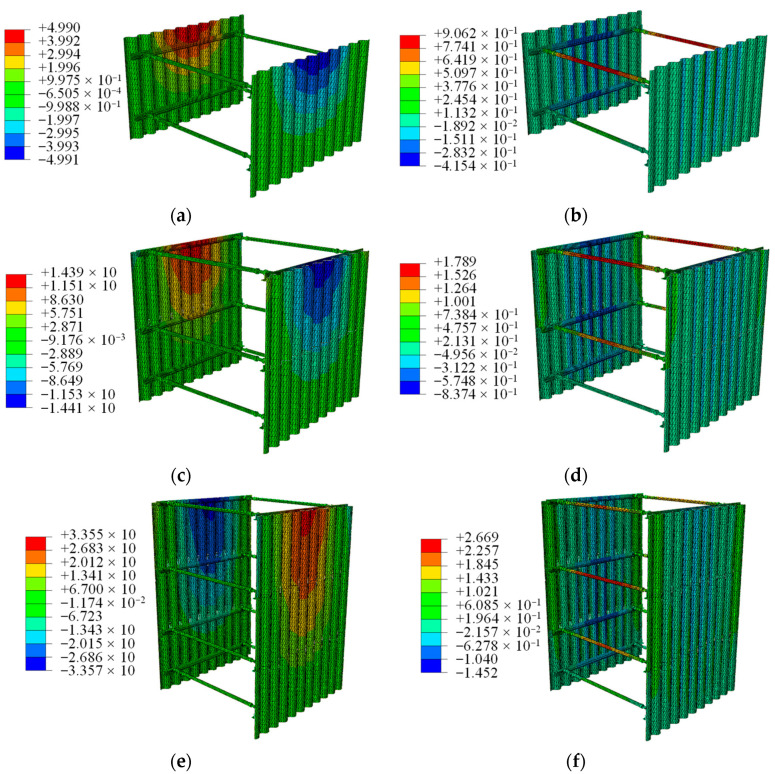
Structure displacement cloud map; (**a**) horizontal deformation in 2 m deep trench; (**b**) vertical deformation in 2 m deep trench; (**c**) horizontal deformation in 3 m deep trench; (**d**) vertical deformation in 3 m deep trench; (**e**) horizontal deformation in 4.5 m deep trench; (**f**) vertical deformation in 4.5 m deep trench.

The displacement of the support structure after applying twice the vehicle load to both sides of the thermal trench is illustrated in Figure 15. Overall, the maximum horizontal displacement of the support structure occurs at the upper portion of the corrugated plate, while the maximum vertical deformation is located at the center of the transverse support.

At trench depths of 2 m, 3 m, and 4.5 m, the maximum horizontal deformation of the structure is 4.9 mm, 14.3 mm, and 33.5 mm, respectively, while the maximum vertical deformation is 0.9 mm, 1.7 mm, and 2.6 mm, respectively. In summary, applying the optimized support structure to trenches with depths of 2 m, 3 m, and 4.5 m results in limited deformation, all within 50 mm.

A comprehensive analysis of the stress and displacement results indicates that the optimized support structure performs well when applied to trenches with depths of 2 m and 3 m. However, when the optimized support structure is applied to a trench with a depth of 4 m, the stress on the angle steel, screw jacks, and pins exceeds the material’s yield limits, potentially leading to structural damage. Consequently, when utilizing the optimized support structure in trenches with a depth of 4 m, it is recommended that no heavy objects be placed on either side of the trench to prevent undue stress and potential failure.

### 5.4. Superiority of Recyclable Non-Embedded Support

The newly developed recyclable, non-embedded depth support structure for thermal pipeline trenches demonstrates several significant advantages that address current challenges in trench construction, particularly when compared to traditional support systems. These benefits are not only theoretical but have practical implications that can significantly improve construction efficiency, safety, and sustainability.

(1) Efficient construction efficiency: The modular design of the support system allows for quick assembly and disassembly, drastically reducing installation time. This feature is especially valuable in urgent repair projects or those with tight deadlines, where rapid deployment is critical. In comparison with conventional systems, which often require heavy machinery and longer setup times, the proposed system offers a more streamlined approach, reducing labor costs and improving overall project efficiency.
(2) Improved flexibility and adaptability: The adjustable screw jack mechanism allows the support system to accommodate various trench depths and soil conditions. This adaptability is a significant advantage over traditional supports, which often lack the flexibility to adjust to different environments. This feature enhances the applicability of the system to a wider range of construction scenarios, from shallow urban trenches to deeper installations in varied terrains, thereby expanding its potential for real-world use.
(3) Cost-effectiveness and economic sustainability: The modular nature of the system promotes standardized production and component reuse, which lowers overall construction and maintenance costs. Unlike traditional systems, which involve significant upfront costs for materials and labor, the proposed support structure reduces both capital expenditures and long-term maintenance costs, providing a more economical solution for trench construction in the urban context.
(4) Environmental sustainability: The recyclable design of the system not only reduces material waste but also helps to minimize carbon emissions, aligning with modern environmental standards in civil engineering. Unlike conventional supports that may involve non-recyclable materials or require extensive equipment for installation and removal, the proposed support system offers a more sustainable alternative, contributing to green construction practices and reducing the carbon footprint of pipeline projects.
(5) Controllable construction period: The ease of installation and disassembly contributes to a shorter construction period, allowing projects to meet tight deadlines without sacrificing quality. This time-saving feature makes the system particularly useful in scenarios where fast-track construction is necessary, such as in urban infrastructure upgrades or emergency repairs. In comparison with traditional systems, which often face delays due to complicated installation processes, the proposed system offers a more agile solution, improving project delivery times.

### 5.5. Discussion

While the results of the prototype test and numerical simulation are promising, several limitations need to be addressed in further research:

(1) The system’s performance was evaluated primarily in trench depths of 2 to 4 m. While the system performed well within this range, caution is required when extending its application to deeper trenches. The stress levels in the 4.5 m trench exceeded the material’s yield strength, which suggests that the system may not be as effective under extreme conditions without further optimization. Future studies should focus on enhancing the structural integrity of the system for deeper applications, perhaps by reinforcing key components or adjusting the modular design to handle higher loads.
(2) The performance was based on idealized soil conditions (sandy soil) obtained from a local in situ test. In practice, soil conditions can vary significantly across different regions, and this variability may affect the performance of the support system. The interaction between soil properties, such as cohesion, compaction, and moisture content, and the support system needs further exploration. Simulating various soil types and environmental conditions in future research will help better understand the system’s performance in diverse settings.
(3) The numerical simulations in this study rely on several assumptions, such as idealized soil conditions and simplified material properties. These assumptions were made to facilitate the modeling process, but they may not fully capture the complexities of real-world scenarios. Future research should focus on analyzing the impact of these assumptions on the system’s performance.

## 6. Conclusions

This study presents a novel recyclable, non-embedded support system for thermal pipeline trenches, addressing the inefficiencies, high costs, and environmental impact associated with traditional support methods. Key findings of the research are as follows:

(1) The proposed support system demonstrated excellent performance in trenches with depths of 2 m and 3 m, maintaining safety and stability under 58 kPa load and rainfall, with maximum stress of −61.51 MPa and deformation of 24 mm. In a 4 m deep trench with an 80 kPa asymmetric load, the system exhibited a maximum stress of 22.27 MPa and deformation of 45 mm, ensuring structural integrity within safe limits.
(2) Optimization of the system, including the addition of screw jacks and adjustments to corrugated plates, significantly improved structural performance by reducing weight and enhancing adjustability without compromising safety.
(3) The system’s modular design enables rapid installation and disassembly, making it highly adaptable to various trench sizes and construction conditions, thus improving construction efficiency and flexibility compared to conventional support methods.

## Figures and Tables

**Table 1:** Design parameters of supporting structure.

Trench	Corrugated Plate	Transverse Support	Bolts	Angle Steel
F2	Wavelength: 230 mm; wave height: 64 mm; thickness: 5 mm.	Diameter: 70 mm; thickness: 6 mm.	Angle steel-corrugated plate: M20; Angle steel- Transverse support: M16.	Width: 75 mm; height: 75 mm; thickness: 10 mm
F3	Wavelength: 300 mm; wave height: 116 mm; thickness: 5 mm.	Diameter: 102 mm; thickness: 6 mm.	Angle steel-corrugated plate: M20; Angle steel- Transverse support: M16.	Width: 100 mm; height: 100 mm; thickness: 10 mm
F4	Wavelength: 381 mm; wave height: 152 mm; thickness: 5 mm.	Diameter: 70 mm; thickness: 6 mm.	Angle steel-corrugated plate: M20; Angle steel- Transverse support: M16.	Width: 125 mm; height: 125 mm; thickness: 10 mm

**Table 2:** Prototype test cases.

Case	Loading Weigh (kPa)	Loading Form	Rainfall
F2	10, 18, 26, 34, 42, 50, 58	Symmetric loading	No
F3	10, 18, 26, 34, 42, 50, 58	Asymmetric loading	No
F2_R	58	Symmetric loading	Yes
F4	10, 30, 80	Asymmetric loading	No

**Table 3:** Deformation of the transverse support (mm).

Test Case	HC-X-1	HC-X-2	HC-S-1	HC-S-2
F2_58	4	3	3	3
F3_58_S	2	3	2	2
F3_58_D	4	5	3	3
F2_R	6	7	5	5
F4_80_S	3	3	2	2
F4_80_D	6	7	4	4

**Table 4:** Stress of the transverse support (MPa).

Case	Structure Stress	Case	Structure Stress	Case	Structure Stress
F2_58	−30.13	F3_58_D	41.57	F4_80_S	22.27
F3_50_S	43.04	F2_R	−61.51	F4_80_D	−13.30

**Table 5:** Soil deformation at the trench bottom.

Test Case	Deformation/mm	Test Case	Deformation/mm
F4_10_S	0	F4_10_D	2
F4_30_S	2	F4_30_D	4
F4_80_S	4	F4_80_D	4

**Table 6:** Optimization design parameters.

Corrugated Plate Parameter	Horizontal Support	Angle Steel	Bolt
Wavelength: 300 mm; wave height: 116 mm; thickness: 5 mm.	Diameter: 70 mm; thickness: 6 mm.	Width: 75 mm; height: 110 mm; thickness: 10 mm	Angle steel-corrugated plate: M20; Angle steel- Transverse support: M16.

## Data Availability

Data is contained within the article.

## References

[1] Barton W., Zohne R., Tabesh A., Sultan F., Najafi M., Rezai S., Heidrick J.W., Mihm M.S. (2019). Case Study from Trenchless Rehabilitation of 60-Inch Residuals Transfer Main at East Side Water Treatment Plant. Proceedings of the Conference on Pipeline Engineering-Concepts in Harmony (PIPELINES).

[2] Shao B., Zhang H., Tang J., Qin T., Bai S., Lei Z., Zuo J., Yu J. (2023). Research on simple support for mechanized construction of trench and shallow foundation pit in soft soil areas. Build. Struct..

[3] Feng T., Zhou K., Zhang J., Chen Z., Peng P. (2024). Study on the Impact of Large-Section Rectangular Pipe Jacking Construction on Existing Pipelines. Eng. Mech..

[4] Jiao N., Wan X., Ding J., Zhang S., Liu J. (2024). Pipeline deformation caused by double curved shield tunnel in soil-rock composite stratum. Geomech. Eng..

[5] Yu S., Lan R., Luo J., Duan Z., Ma S. (2021). An Investigation of Effects and Safety of Pipelines due to Twin Tunneling. Adv. Civ. Eng..

[6] Zhang X., Liang C., Huang S., Xu Y. (2023). Gas Pipeline Response to Underlying Straight-Wall Arch Tunnel Construction. Buildings.

[7] Dang L., Zhang L., Sun D., Sung W.P., Chen R. (2011). The optimization of design and construction for an internal supporting structure. International Conference on Green Building, Materials and Civil Engineering (GBMCE 2011).

[8] Wang S., Zheng Z., Mu Z., Zhang J., Zhang X.D., Li H.N., Feng X.T., Chen Z.H. (2013). Influence of Pit Excavation Supported by Soil-nailing Wall on Adjacent Buried Pipelines. Proceedings of the 2nd International Conference on Civil Engineering and Transportation (ICCET 2012).

[9] Zhang Z., Zhang M., Zhao Q. (2015). A simplified analysis for deformation behavior of buried pipelines considering disturbance effects of underground excavation in soft clays. Arab. J. Geosci..

[10] Zhang J., Xie R., Zhang H. (2018). Mechanical response analysis of the buried pipeline due to adjacent foundation pit excavation. Tunn. Undergr. Space Technol..

[11] Jiang N., Zhu B., He X., Zhou C., Luo X., Wu T. (2020). Safety assessment of buried pressurized gas pipelines subject to blasting vibrations induced by metro foundation pit excavation. Tunn. Undergr. Space Technol..

[12] Wu B., Ge C., Li P., Yang M., Li L. (2024). Influence of Deep Foundation Pit Excavation on Adjacent Pipelines: A Case Study in Nanjing, China. Appl. Sci..

[13] Xing T., Liu H., Zheng J., Yu X., Li Y., Peng H. (2024). Study on the Effect of Anchor Cable Prestress Loss on Foundation Stability. Appl. Sci..

[14] Tao K., Wang Q., Yue D. (2024). Data Compression and Damage Evaluation of Underground Pipeline With Musicalized Sonar GMM. IEEE Trans. Ind. Electron..

[15] Oda K., Kaneko S., Kishi S. Design Method of Pipeline in Shield Tunnel against Fault Displacement. Proceedings of the Lifelines Conference (Lifelines).

[16] Ouyang W.-H., Yang Y., Wan J.-H., Liu S.-W. (2020). Second-Order Analysis of Steel Sheet Piles by Pile Element Considering Nonlinear Soil-Structure Interactions. Adv. Steel Constr..

[17] Dhutti A., Dhutti A., Malo S., Marques H., Balachandran W., Gan T.-H. (2021). Numerical Modelling of Ultrasonic Guided Wave Propagation and Defect Detection in Offshore Steel Sheet Piles. Appl. Sci..

[18] Gao L., Xu Z., Wang Q., Zhang Z., Li P. (2021). Model Test Study on Deformation of Snowflake Shaped Steel Sheet Pile Based on OFDR. Sensors.

[19] Jiang Y., Guo F., Wang W., Yang G., Yue J., Huang Y. (2023). Stability Study of a Double-Row Steel Sheet Pile Cofferdam Structure on Soft Ground. Water.

[20] Wang L., Zhang C., Cui G., Wang X., Ye Z. (2022). Study on the performance of the new composite thermal insulation lining for the railway operational tunnel in cold regions. Case Stud. Therm. Eng..

[21] (2012). Code for Desigin of Building Foundation.

